# Urothelial Superior Vena Cava Syndrome with Limited Response to Radiation Therapy

**DOI:** 10.1155/2015/513685

**Published:** 2015-11-08

**Authors:** Nishan Bingham, H. James Wallace III, Joanne Monterroso, Claire Verschraegen, Brenda L. Waters, Christopher J. Anker

**Affiliations:** ^1^University of Vermont College of Medicine, 89 Beaumont Avenue, Burlington, VT 05405, USA; ^2^Division of Radiation Oncology, University of Vermont Cancer Center, 111 Colchester Avenue, Mailstop 301SH2, Burlington, VT 05401, USA; ^3^Division of Hematology Oncology, University of Vermont Cancer Center, 89 Beaumont Avenue, Suite E-214, Burlington, VT 05405, USA; ^4^Department of Pathology and Laboratory Medicine, University of Vermont Medical Center, 111 Colchester Avenue Main Campus, East Pavilion, Burlington, VT 05401, USA

## Abstract

Radiation therapy (RT) is the standard of care for cases of superior vena cava (SVC) syndrome secondary to metastatic adenopathy. Histologies vary in radiosensitivity and response time, making alternative therapies such as chemotherapy and/or intravenous stenting preferable alternative options for certain diagnoses. Metastatic urothelial carcinoma is a particularly rare cause of SVC syndrome with only 3 cases reported in the literature. Consequently, optimal management remains challenging, particularly in cases of high tumor burden. Here we present a case of highly advanced metastatic urothelial cancer with SVC syndrome and tracheal compression. The patient started urgent RT but expired midway through her treatment course due to systemic progression of disease, requiring SVC and tracheal stenting. The authors review the literature including discussion of the few other known cases of SVC syndrome due to urothelial carcinoma and a review of this histology's response to RT. This experience suggests, that in cases of SVC syndrome with widespread advanced disease, stenting and chemotherapy with or without RT may be the most important initial treatment plan, depending on goals of care.

## 1. Introduction

Instances of metastatic urothelial carcinoma causing superior vena cava (SVC) syndrome are extremely rare. Average survival of SVC syndrome is six months but varies with histology [1]. For most cases, urgent treatment with radiation therapy (RT) with or without stenting is the standard of care, while some histologies are best treated with chemotherapy [2, 3]. Although RT has proven beneficial in symptom palliation and disease control for urothelial cancer, there remain very limited data regarding its utility in cases of high tumor burden. Here we report a patient with widely metastatic bladder cancer causing SVC syndrome and tracheal compression. The disease was unresponsive to RT and steroids and required stenting. However within 1.5 weeks the patient died from extensive systemic tumor burden. This case underscores the challenges of selecting therapy for urothelial SVC syndrome as well as the need for further characterization of the timeframe of urothelial carcinoma's response to RT.

## 2. Case Report

**Figure 1:**
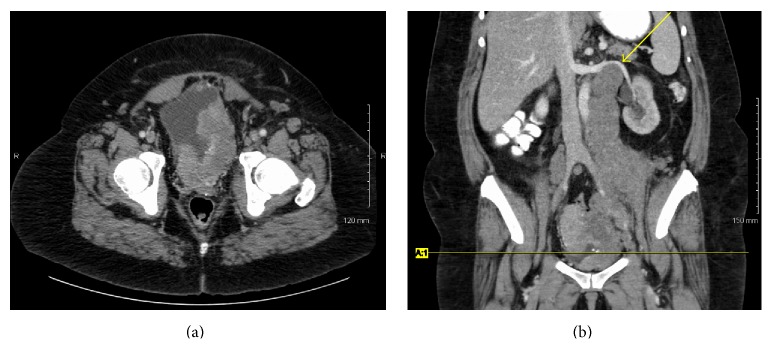
Axial (a) and coronal (b) pelvic CT scan images showing bladder wall thickening and retroperitoneal masses displacing left renal vein (arrow). The axial image in (a) is at the level of the horizontal line noted in (b).

**Figure 2:**
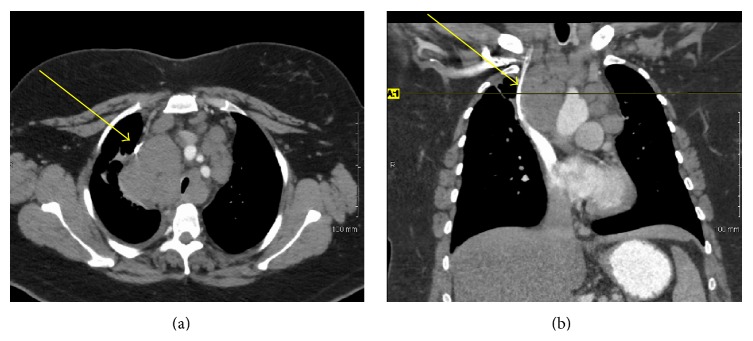
Axial (a) and coronal (b) chest CT images showing mediastinal lymphadenopathy compressing the SVC (arrows) and trachea. The axial image in (a) is at the level of the horizontal line noted in (b).

**Figure 3:**
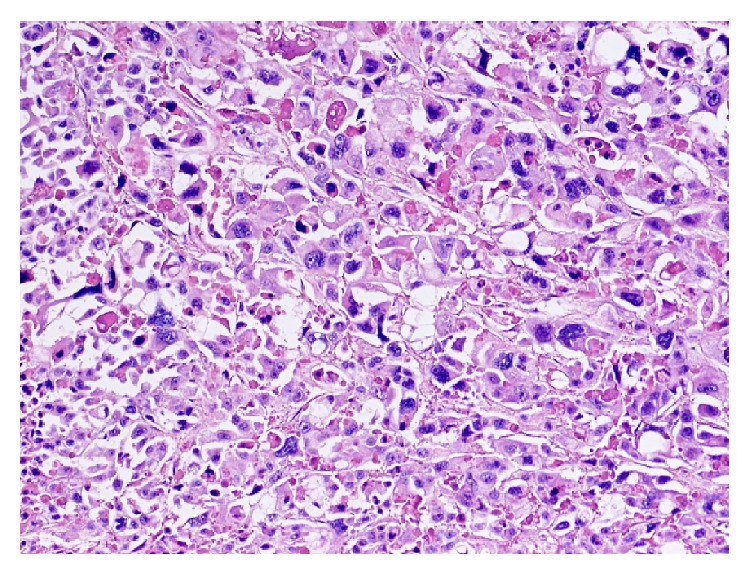
The supraclavicular lymph node was entirely effaced by sheets of highly pleomorphic epithelioid cells with enlarged hyperchromatic nuclei, focal cytoplasmic vacuolization, and areas (not pictured here) of necrosis.

**Figure 4:**
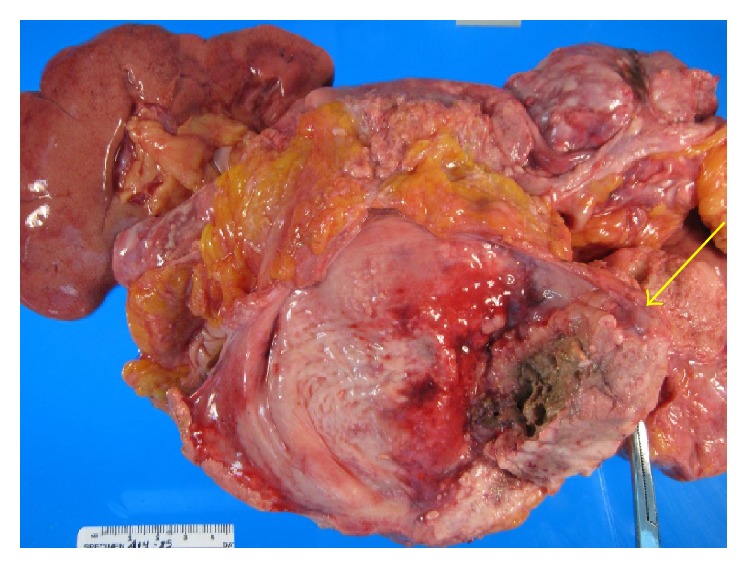
Tumor mass (arrow), left side of the bladder. Extensive metastases to lymph nodes with normal urothelium.

**Figure 5:**
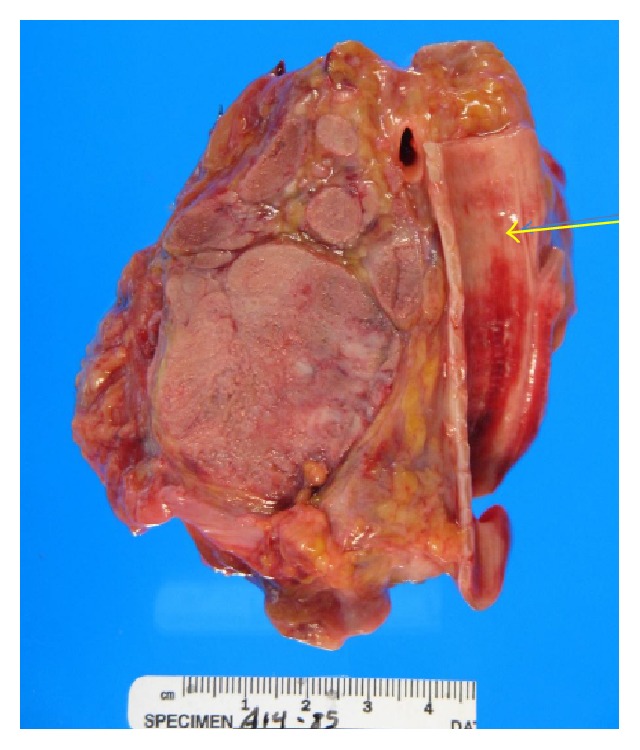
Massive adenopathy anterior to trachea (arrow).

**Figure 6:**
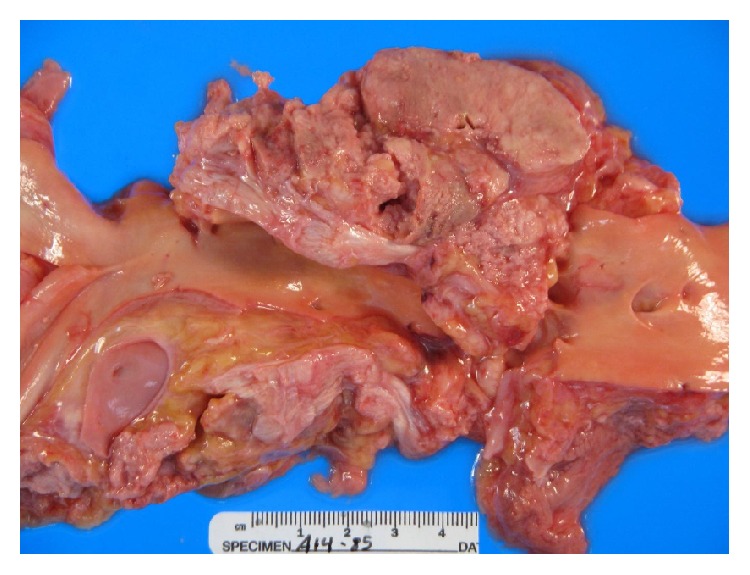
Lymphadenopathy along entire length of aorta by tumor.

**Figure 7:**
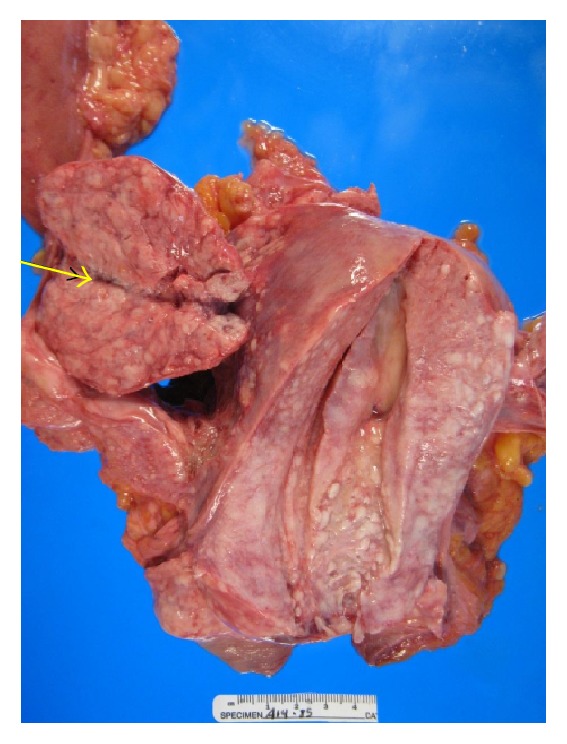
Massive enlargement of ovary (arrow) and uterus from extensive tumor infiltration.

**Figure 8:**
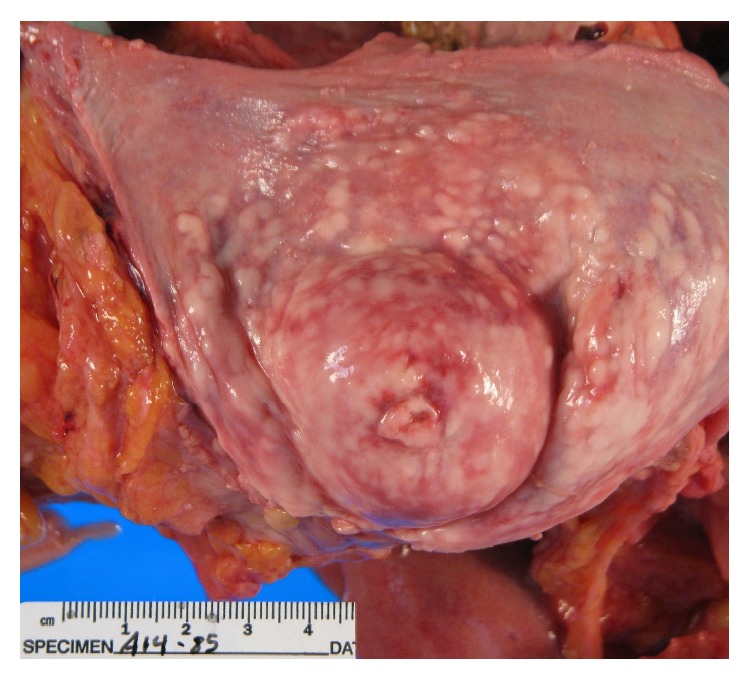
Tumor infiltrating surface of exocervix and vagina.

A 48-year-old woman presented to her primary care physician with dysuria and urinary frequency unresponsive to antibiotics. One month later she presented to the emergency room with worsening flank pain and new-onset dyspnea, cough, hoarseness, and fatigue. She had no history of smoking or chronic bladder irritation, nor did she report any occupational exposure to known chemical carcinogens. Exam was notable for tachycardia, right eye ptosis and miosis, cervical edema, bilateral supraclavicular lymphadenopathy, and stridor. Urine cytology showed high grade malignant cells. Flexible cystoscopy showed a solid mass in the left mid-lateral wall. Pelvic (Figure 1) and chest (Figure 2) computed tomography (CT) scans showed widespread disease with a thickened, calcified bladder wall, bulky retroperitoneal masses, and extensive lymphadenopathy compressing the SVC and trachea. Supraclavicular node biopsy (Figure 3) supported the diagnosis of stage IV urothelial carcinoma with GATA3 positive, cytokeratin 7 (CK 7) positive, and cytokeratin 20 (CK 20) negative immunohistochemistry. She began dexamethasone (4 mg every 6 hours) and RT (30 Gy in 10 fractions planned) to the mediastinum for SVC and tracheal decompression. However, over several days, her dyspnea worsened, and she developed tumor lysis syndrome with hypertension and acute kidney injury. Persistent tracheal compression prompted stenting and ICU transfer. On the 7th day, respiratory decompensation required intubation with tracheostomy for bilateral vocal cord paralysis from nerve compression. RT was held after only 18 Gy and chemotherapy was not administered because of rapid deterioration. She died on hospital day 11 shortly after the family withdrew care. Autopsy showed a large, expansile tumor mass originating in the bladder wall (Figure 4), massive tumor adenopathy anterior to the trachea (Figure 5), along the aorta (Figure 6), and metastases to lung, liver, spleen, omentum, ovaries/uterus (Figure 7), and cervix/vagina (Figure 8).

## 3. Discussion

RT is considered standard of care in cases of malignancy causing compression of the SVC and trachea because it generally provides a prompt and durable response. Exceptions include small-cell lung cancer and lymphoma, which usually respond to chemotherapy within 72 hours. Newer techniques from interventional radiology such as stenting provide alternative management options, but there are no randomized trials comparing these techniques.

While 96–100% of ovarian adenocarcinomas are CK 7+/CK 20−, compared with only 11–63% of bladder cancers [4, 5], urothelial carcinomas display immunohistochemical GATA3 positivity whereas ovarian cells do not [6, 7]. GATA3 is a sensitive urothelial immunomarker staining in 86% of patients, and it is also specific as the only additional carcinomas it stains positive for are breast (94%) and to a much lower frequency endometrial (2%). Moreover, the finding at autopsy of tumor appearing to originate from the bladder was consistent with a primary urothelial carcinoma. SVC syndrome from a primary bladder cancer is extremely rare, with only three cases reported in the literature. One case was treated with 40 Gy of RT over 20 days causing grade 3 esophagitis, but no tumor response. Treatment was switched to paclitaxel, gemcitabine, and cisplatin which decreased the offending tumor size by 90%. The patient died 10 months later due to bleeding from a brain metastasis [8]. None of the other cases survived more than one month, although one had initial improvement with chemoradiation [9–11].

Currently, the standard of care for metastatic urothelial carcinoma is cisplatin-based combination chemotherapy. The combination of cisplatin and gemcitabine has shown improved tolerability compared to that of methotrexate, vincristine, adriamycin, and cisplatin [12]. Overall, poor performance status and the presence of bone or visceral metastases are the most reliable prognostic indicators of a poor response to treatment, with very few patients living beyond six years [12].

Experience with SVC syndrome from urothelial carcinoma remains extremely limited, with little to no follow-up. In rapidly progressive disease, immediate stenting followed by chemotherapy or chemoradiation may be reasonable treatment options. Palliative RT for bleeding has been shown to induce hemostasis within two weeks in 72% of patients with muscle-invasive disease who develop gross hematuria, suggesting that such a time frame may be necessary to observe significant benefit from RT [13]. Stereotactic hypofractionated techniques may present an alternative treatment strategy that could improve long-term local control. In a report detailing treatment outcomes for brain metastases from bladder cancer, the one patient treated with stereotactic radiosurgery survived over one year with good local control, while five of eleven patients receiving whole brain radiation died during treatment [14].

Targeted treatment decisions should be based on clinical signs and symptoms, as well as overall treatment goals. Local therapies may significantly improve quality of life and survival especially when SVC syndrome is a life threatening issue. For urothelial carcinoma, systemic therapy may also be necessary for treatment of SVC syndrome. Interestingly, in the future, further characterization of radio- and chemosensitivities for genomic subsets of urothelial carcinoma may provide rapid histologic and genomic characterization that could clarify optimal therapy in both acute and long-term settings [15–17].
